# HEV Occurrence in Waste and Drinking Water Treatment Plants

**DOI:** 10.3389/fmicb.2019.02937

**Published:** 2020-01-14

**Authors:** Enric Cuevas-Ferrando, Walter Randazzo, Alba Pérez-Cataluña, Gloria Sánchez

**Affiliations:** ^1^Department of Preservation and Food Safety Technologies, Instituto de Agroquímica y Tecnología de Alimentos – Consejo Superior de Investigaciones Científicas (IATA-CSIC), Valencia, Spain; ^2^Department of Microbiology and Ecology, University of Valencia, Valencia, Spain

**Keywords:** Hepatitis E virus, wastewater, drinking water, water quality, RT-qPCR, occurrence

## Abstract

Hepatitis E virus (HEV), particularly zoonotic genotype 3, is present in environmental waters worldwide, especially in industrialized countries. Thus, monitoring the presence of HEV in wastewater treatment plants (WWTPs) is an emerging topic due to the importance of reusing water on a global level. Given the limited data, this study aimed to monitor the occurrence of HEV in influent and effluent water in waste- and drinking-water treatment plants (WWTPs and DWTPs). To this end, different procedures to concentrate HEV in influent and effluent water from WWTPs and DWTPs were initially evaluated. The evaluated procedures resulted in average HEV recoveries of 15.2, 19.9, and 16.9% in influent, effluent, and drinking water samples, respectively, with detection limits ranging from 10^3^ to 10^4^ international units (IU)/L. Then, a one-year pilot study was performed to evaluate the performance of the selected concentration method coupled with three RT-qPCR assays in influent and effluent water samples from four different WWTPs. HEV prevalence in influent water varied based on both the RT-qPCR assay and WWTP, while HEV was not detected in effluent water samples. In addition, HEV prevalence using only RT-qPCR3 was evaluated in influent (*n* = 62) and effluent samples (*n* = 52) from four WWTPs as well as influent (*n* = 28) and effluent (*n* = 28) waters from two DWTPs. The present study demonstrated that HEV circulated in the Valencian region at around 30.65% with average concentrations of 6.3 × 10^3^ IU/L. HEV was only detected in influent wastewater samples, effluent samples from WWTPs and influent and effluent samples from DWTPs were negative. However, given that the infective dose in waterborne epidemics settings is not yet known and the low sensibility of the assay, unfortunately, no direct conclusion could be achieved on the risk assessment of environmental contamination.

## Graphical Abstract

Method performance and HEV prevalence in waters.

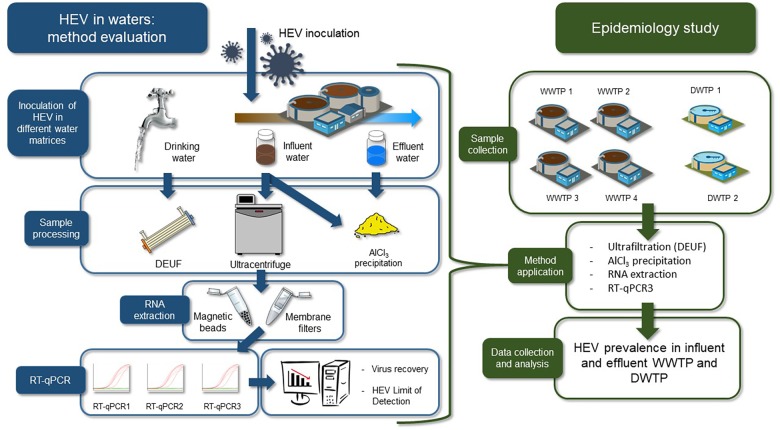

## Introduction

Hepatitis E virus (HEV) is a human enteric virus that mainly causes self-limiting acute viral hepatitis. According to the World Health Organization, 20 million cases of hepatitis E and 44,000 deaths occur worldwide every year^1^. HEV is an emerging foodborne pathogen (Harrison and DiCaprio, 2018), and the incidence of confirmed cases in the European Union has steadily increased over the last decade (Kupferschmidt, 2016; Ricci et al., 2017).

Hepatitis E infections are caused by a small (27–34 nm), positive-sense, single-stranded RNA virus (approx. 7.2 kb size) that belongs to the *Hepeviridae* family (Sooryanarain and Meng, 2019; Van der Poel and Rzezutka, 2019). HEV is excreted in feces as non-enveloped virions but circulates in the blood in a membrane-associated, quasi-enveloped form (Yin et al., 2016). HEV is classified into eight genotypes, of which genotype 1 (G1) and G2 are specific to humans. HEV G3, G4, and G7 are zoonotic genotypes that infect humans and animals and have been isolated in different animal species, especially in pigs (Van der Poel, 2014; Sooryanarain and Meng, 2019). The different HEV genotypes have different geographical distributions^2^. For example, HEV G1 and G2 are predominantly transmitted via the fecal-oral route in Asia, Africa, and Central America, usually through the consumption of contaminated drinking water (Khuroo et al., 2016; Van der Poel and Rzezutka, 2019). In contrast, HEV G3 and G4 are endemic in industrialized countries and transmitted primarily via the consumption of animal meats or direct contact with infected animals (Sooryanarain and Meng, 2019).

Hepatitis E virus transmission to humans through water has been largely demonstrated for HEV G1 and G2, primarily in developing countries, but transmission is also suspected for the zoonotic genotypes since HEV G3 and G4 have been detected in different types of environmental waters (Miura et al., 2016; Haramoto et al., 2018; Fenaux et al., 2019; Van der Poel and Rzezutka, 2019). Given the authorities’ concerns, several surveillance studies conducted in different geographic regions have assessed the presence of HEV in urban wastewater with highly variable occurrence (Fenaux et al., 2019). However, few studies have focused on effluent wastewater or drinking water (Fenaux et al., 2019; Purpari et al., 2019; Van der Poel and Rzezutka, 2019). In addition, available data must be interpreted with caution due to the lack of standardized HEV detection procedures and the substantial differences among studies in terms of volume of samples, concentration methods, and RT-qPCR (Fenaux et al., 2019).

To overcome these challenges, this study initially evaluated the performances of different concentration methods, RNA extraction kits, and RT-qPCR protocols in detecting and quantifying HEV in influent and effluent wastewater samples as well as in drinking water samples (Graphical Abstract). After method evaluation, the presence of HEV was monitored in influent and effluent waters from four municipal wastewater treatment plants (WWTPs) and two drinking water treatment plants (DWTPs) in the metropolitan region of Valencia (Spain).

## Materials and Methods

### Virus Strains

Fecal sample containing HEV genotype 3f was used in the study. Fecal sample (10% wt/vol) was suspended in phosphate-buffered saline (PBS) containing 2 M NaNO_3_ (Panreac), 1% beef extract (Conda), and 0.1% Triton X-100 (Thermo Fisher Scientific) (pH 7.2). The mix was then vigorously vortexed and centrifuged at 1,000 × *g* for 5 min to obtain a final 10% (wt/vol) fecal suspension. The supernatant was stored at −80°C in aliquots. The first WHO international standard for HEV nucleic acid amplification technique (NAT)-based assays (code 6329/10) was purchased from Paul-Ehrlich-Institut (Germany). This standard corresponds to HEV genotype 3a positive plasma measured in international units (IU) and containing 250,000 IU/mL and it was used for RT-qPCR quantification, as detailed below (Baylis et al., 2013). Mengovirus (MgV) vMC_0_ (CECT 100000) was used as a process control.

### Sampling Sites

**FIGURE 1:**
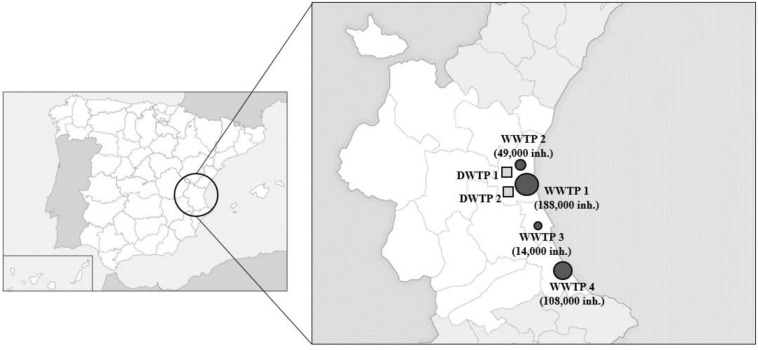
Map of the sampling locations. WWTP, wastewater treatment plant (squares); DWTP, drinking water treatment plants (circles). Symbols are sized according to the number of inhabitants.

Influent and effluent water samples were collected from four WWTPs and two DWTPs located in the Valencian region, Spain (Figure 1). The collected samples were transferred to the laboratory immediately, and subsequently concentrated as described below.

### Concentration Procedure Comparison in Influent Wastewater

Influent water samples collected from WWTP1 were artificially inoculated with 5 log IU/L of HEV and 7 log PCRU/L of MgV, spiked as process control.

Initially, the performance of two concentration methods was evaluated: an ultracentrifugation-based method (referred as UC) and an aluminum hydroxide adsorption-precipitation method (referred as Al). For UC method, 35 mL of influent water were centrifuged at 141,000 × *g* for 2 h 30 min at 4°C. The pellet was then incubated on ice for 30 min with 5 mL of 0.25 N glycine buffer (pH 9.5) and then the solution neutralized with 19 mL of PBS. Suspended solids were removed by centrifugation at 12,000 × *g* for 15 min. Viruses were finally recovered by ultracentrifugation at 505,000 × *g* for 1 h at 4°C and subsequently eluted in 1 mL of PBS (Rodríguez-Díaz et al., 2009).

For Al method, 35 mL of influent water were adjusted to pH 6.0 and Al(OH)_3_ precipitate formed by adding 1 part 0.9N AlCl_3_ solution to 100 parts of sample. The pH was readjusted to 6.0 and sample mixed using an orbital shaker at 150 rpm for 15 min at room temperature. Then, viruses were collected by centrifugation at 1,700 × *g* for 20 min. The pellet was resuspended in 1.75 mL of 3% beef extract pH 7.4, and samples were shaken for 10 min at 150 rpm. Concentrate was recovered by centrifugation at 1,900 × *g* for 30 min and pellet resuspended in 1 mL of PBS (AAVV, 2018; Randazzo et al., 2019) and stored at −80°C. Experiments were performed in duplicate.

### Detection Limit in Influent and Effluent Wastewater

The limit of detection (LoD_95__%_) was obtained by artificially inoculating HEV at 5, 4, 3, and 2 log IU/L in 35 mL for influent water or in 200 mL for effluent water.

Samples were spiked with MgV (7 log PCRU/L) as a process control. Then, virus particles were concentrated by the previously described Al method and RNA extracted using two kits and analyzed by RT-qPCR1 and RT-qPCR2 (detailed below). For each method and contamination level, a PBS sample without influent or effluent water were included to assess potential matrix effects. Experiments were performed in duplicate by concentrating two independent samples for each condition tested.

### Concentration Procedure Comparison and Detection Limit in Drinking Water

Drinking water samples (20 L) were artificially inoculated with HEV at 7, 6, 5, and 4 log IU/L. In addition, MgV was spiked and used as process control. HEV primary concentration was performed by a Dead End Hollow Fiber Ultrafiltration (DEUF) using single-use Rexeed-25A dialysis filters (Asahi Kasei Medical Co., Ltd.) with a molecular mass cutoff of 30 kDa, a surface area of 2.5 m^2^, a fiber inner diameter of 185 μm and a priming volume of 137 mL (Borgmästars et al., 2017). A peristaltic pump (model FH100, Thermo Fisher Scientific) was used for all experiments.

In brief, the Rexeed-25A filters were blocked with 6.25% fetal bovine serum by circulating the blocking solution for 5 min followed by 2 h incubation at room temperature. Afterward, filter was properly assembled and flushed with 1 L of sterile water at 2,900 mL/min and then with the 20 L of inoculated drinking water samples. Subsequently, filter was assembled for a back-flush elution with 500 mL of sterile water supplemented with 0.001% Antifoam, 0.01% NaPP, and 0.01% Tween 80.

Two different approaches were evaluated for secondary concentration: a precipitation with polyethylene glycol (PEG) and a centrifuge filtration procedure by Amicon^®^ Ultra-15 tubes (Merck Millipore Ltd.). For PEG precipitation, 300 mL of concentrate were transferred to two 250 mL centrifugation tubes, 150 mL of eluate for each tube. Then, 2 g of beef extract (Laboratorio Conda) were added into each tube and shaken until completely dissolved. Then, 50 mL of PEG/NaCl 5× were added and incubated overnight at 4°C in an orbital shaker at 150 rpm. Finally, the samples were centrifuged at 10,000 × *g* for 30 min and resulting pellets resuspended in 1 mL PBS.

For secondary concentration by centrifuge filtration, 15 mL volume was added to Amicon^®^ Ultra-15 tube and concentrated via centrifugation at 4,000 × *g* for 15 min. This step was repeated three times using the same ultrafilter for a total of 45 mL sample processed. Then the concentrated viruses were recovered in 1 mL PBS. The viral concentrates were stored at −80°C until further processed. Experiments were performed in duplicate by concentrating two independent samples for each condition tested.

### RNA Extraction and RT-qPCR Assays

Two different commercial extraction kits were used for RNA extraction. The extraction using the NucleoSpin^®^RNA virus kit (Macherey-Nagel GmbH & Co.) (referred as MN) was performed according to the manufacturer’s instructions with some modifications. Briefly, 150 μL of each concentrated sample was mixed with 25 μL Plant RNA Isolation Aid (Ambion) and 600 μL of lysis buffer from the NucleoSpin^®^ RNA virus kit and subjected to pulse-vortexing for 1 min. Afterward, the homogenate was centrifuged for 5 min at 10,000 × *g* to remove the debris. The supernatant was subsequently processed according to the manufacturer’s instructions. An additional extraction was carried out using the NucliSENS^®^ miniMAG^®^ system (BioMérieux SA) (referred as NS) and according to manufacturer instructions. In particular, the sample volume was 500 μL and the elution volume was 100 μL. Resultant RNA was analyzed using the RNA UltraSense One-Step kit (Invitrogen SA) and RT-qPCR performed as described in Schlosser et al. (2014) for HEV (referred as RT-qPCR1) and as in ISO 15216-1:2017 for MgV (Supplementary Table 5). For both RT-qPCR assays, undiluted and 1/10 diluted RNA was tested to check for RT-qPCR inhibitors.

Moreover, RNAs were also quantified using the ceeramTOOLS^®^ Hepatitis E Virus Detection KHEV commercial kit (BioMérieux SA) (referred as RT-qPCR2) provided with an internal amplification control.

In all experiments, all samples were run in duplicate and different controls were used, including negative process, extraction and RT-qPCR controls, and controls for extraction efficiency.

Hepatitis E virus was quantified by plotting the quantification cycles (Cqs) to an external standard curve built with the International Standard WHO HEV RNA (code 6329/10). Moreover, extraction efficiencies were calculated and used as quality assurance parameters according to ISO 15216-1:2017 (2017).

### Analysis of Naturally Contaminated Influent and Effluent Wastewater

A total of 62 influent and 52 effluent wastewater samples were investigated for the occurrence of HEV as hereafter detailed.

Initially, influent (*n* = 32) and effluent (*n* = 32) water samples were collected from four municipal WWTPs located in the Valencian region (eastern Spain), from May 2018 to March 2019 (Figure 1). Two-hundred milliliters of influent and effluent water samples were processed using the Al procedure. Mengovirus was used as process control. RNA extraction was performed using the NucleoSpin^®^ RNA Virus kit (MN kit) and HEV RNA quantified by both RT-qPCR1 and RT-qPCR2. In addition, RNA samples were analyzed by a third RT-qPCR assay (referred as RT-qPCR3, Supplementary Table 5; Girón-Callejas et al., 2015). Additional influent (*n* = 30) and effluent (*n* = 20) samples were further collected in June, August, and October 2018 and from April 2019 to August 2019 and analyzed by RT-qPCR3 only.

### Analysis of Drinking Water Samples

A total of 28 influent and 28 effluent water samples were collected from two municipal DWTPs (Figure 1) in October and November 2018. The samples were maintained under refrigeration (4°C) for transportation and processed within 24 h. Water samples (20 L) were dechlorinated with sodium thiosulphate (10% wt/vol) after collection, added with mengovirus and concentrated using the Rexeed-25A filters and PEG precipitation, as detailed above. Resultant RNA was extracted by the NucleoSpin^®^ RNA Virus kit (MN kit) and detected by RT-qPCR3.

### Statistical Analysis

Results were statistically analyzed and significance of differences was determined on the ranks with a one-way analysis of variance (ANOVA) and Tukey’s multiple comparison tests. In all cases, a value of *p* < 0.05 was deemed significant. Spearman’s rank-order correlation coefficient (ρ_S_) was determined between inhabitants and HEV positive samples by using Statistica software (StatSoft Inc., Tulsa, OK, United States). The estimated probability of detection with 95% confidence (LoD_95__%_) was calculated by using the PODLOD calculation program (version9) (Wilrich and Wilrich, 2009) for all water samples.

### Ethics Statement

Fecal samples were collected at Hospital Clínico Universitario de Valencia (Valencia, Spain). The study was approved by the Comisión de Ética en Investigación Experimental of the University of Valencia (Spain), in accordance with the World Medical Association’s Declaration of Helsinki and the relevant European and Spanish guidelines and regulations.

## Results and Discussion

### Detection Limit and Efficiency of the Procedure to Concentrate HEV in Influent Water

One major limitation in understanding HEV transmission in contaminated waters is the lack of standardized and validated methods (Ricci et al., 2017). Thus, to provide data on the performance of the HEV detection methods in environmental waters, an ultracentrifugation-based protocol (UC) was compared to an aluminum precipitation procedure (Al) using artificially inoculated influent water samples. The mean HEV recoveries obtained with the UC concentration procedure ranged from 7.98 to 16.83% using MN kit and from 10.24 to 55.08% using NS kit. The Al procedure resulted in mean HEV recovery values ranging from 7.00 to 20.54% and from 10.18 to 90.19% using MN and NS kits, respectively (Table 1).

**TABLE 1:** Performance of concentration methods (ultracentrifugation and aluminum precipitation), RNA extraction kits and RT-qPCR assays for HEV detection in artificially inoculated influent water samples.

**Extraction kit**	**RT-qPCR**	**Ultracentrifugation**		**Aluminum precipitation**	
		**Mean HEV recovery**	**Mean mengovirus**	**Mean HEV recovery**	**Mean mengovirus**
		**(min–max) (%)**	**recovery (%)**	**(min–max) (%)**	**recovery (%)**
MN	RT-qPCR1	16.83*A*(13.33−21.68)	13.76 ± 4.59A	20.54*A*(17.06−24.10)	13.67 ± 2.4A
	RT-qPCR2	7.98*A*(7.75−8.30)		7.00*A*(5.45−8.58)	
NS	RT-qPCR1	55.08*A*(49.24−60.84)	23.31 ± 2.46A	90.19*AB*(84.16−96.22)	54.45 ± 17.06B
	RT-qPCR2	10.24*A*(8.95−12.64)		10.18*A*(8.54−11.82)	

*MN: NucleoSpin^®^RNA virus kit (Macherey-Nagel GmbH & Co.). NS: NucliSENS^®^ miniMag^®^ system (BioMérieux SA). RT-qPCR1: Schlosser et al., 2014. RT-qPCR2: ceeramTOOLS^®^ Hepatitis E Virus Detection KHEV kit (BioMérieux SA). Within each column, different letters denote significant differences among methods (*P* < 0.05).*

The Al procedure was selected for the determination of LoD_95__%_ since an ultracentrifuge is not required. To determine LoD_95__%_, influent water was artificially inoculated with MgV together with four levels of HEV and samples concentrated according to the Al procedure. RNA extraction from concentrates was performed using MN and NS kits and subsequently analyzed by RT-qPCR1 and RT-qPCR2.

**FIGURE 2:**
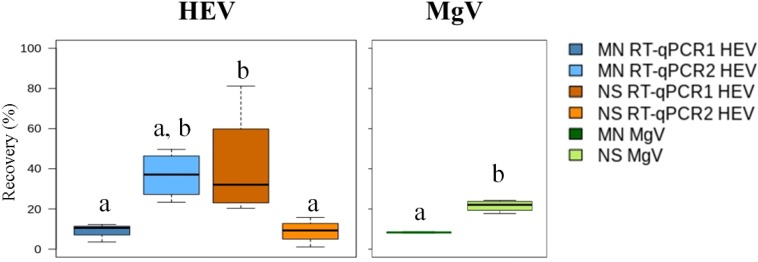
Median HEV recovery (%) in influent water samples using the aluminum protocol and comparing two extraction kits and two RT-qPCRs assays. MN: NucleoSpin^®^RNA virus kit (Macherey-Nagel GmbH & Co.); NS: NucliSENS^®^ miniMag^®^ system (BioMérieux SA); RT-qPCR1: Schlosser et al., 2014; RT-qPCR2: ceeramTOOLS^®^ Hepatitis E Virus Detection KHEV kit (BioMérieux SA). Within each virus, different letters denote significant differences among methods (*P* < 0.05).

The mean HEV recovery values obtained using the MN and NS kits ranged from 8.81 to 36.8% and from 8.90 to 41.45%, respectively (Figure 2 and Supplementary Table 1), and no statistically significant differences were observed (*P* > 0.05). On average, LoD_95__%_ was 2.9 × 10^5^ IU/L for MN kit and 2.2 × 10^6^ IU/L for NS kit, calculated according to Wilrich and Wilrich (2009). Accordingly, LoD_95__%_ increased approximately 10-fold when NS was compared to MN extraction procedure. Overall, the MN kit combined with RT-qPCR1 provided the best LoD_95__%_, which was similar to or slightly higher than those previously reported for other enteric viruses in influent waters (approx. 10^4^–10^5^ genome copies/L) (Nordgren et al., 2009; Randazzo et al., 2019).

The MgV recovered using the MN and NS kits ranged from 7.92 to 8.72% (8.34% mean) and from 17.76 to 24.29% (21.56% mean), respectively (Figure 2 and Supplementary Table 1). These results support previously reported MgV recoveries in influent waters (Miura et al., 2016). Because only 35 mL of sample are needed for the analysis and ultracentrifugation is not required, the procedure is a potential alternative method for routine influent water screening.

### Detection Limit and Efficiency of the Al Procedure to Concentrate HEV in Effluent Water

**FIGURE 3:**
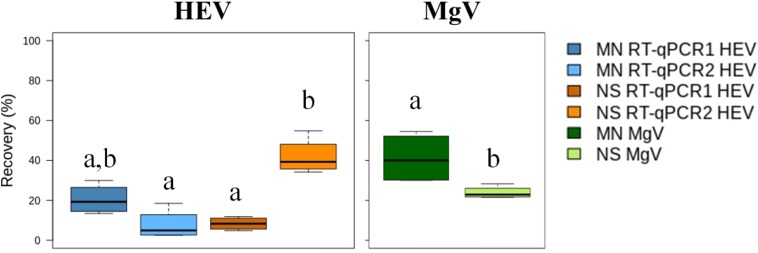
Median HEV recovery (%) in effluent water samples using the aluminum protocol and comparing two extraction kits and two RT-qPCRs assays. MN: NucleoSpin^®^RNA virus kit (Macherey-Nagel GmbH & Co.); NS: NucliSENS^®^ miniMag^®^ system (BioMérieux SA); RT-qPCR1: Schlosser et al., 2014; RT-qPCR2: ceeramTOOLS^®^ Hepatitis E Virus Detection KHEV kit (BioMérieux SA). Within each virus, different letters denote significant differences among methods (*P* < 0.05).

Few studies over the last decade have assessed the presence of HEV in effluent water samples due in part to the lack of validated procedures (Fenaux et al., 2019). Therefore, the performance of the Al concentration method was analyzed using effluent water samples that were collected downstream from WWTP1 and artificially spiked with four levels of HEV and with MgV, as process control. The MN and NS extraction kits and RT-qPCR1 and RT-qPCR2 were used for sample processing. Viral recovery and HEV LoD_95__%_ were determined and the results are shown in Figure 3 and Supplementary Table 2.

MgV recoveries using the MN and NS kit ranged from 30.08 to 54.50% (41.17% mean) and from 21.52 to 28.32% (23.90% mean), respectively, which are slightly higher than the 8–13% recovery rates of cross-flow ultrafiltration reported previously (Miura et al., 2016). The mean recovery of HEV ranged from 8.33 to 30.01% using the MN kit and from 7.72 to 41.90% with the NS kit. LoD_95__%_ was 1.25 × 10^4^ IU/L regardless of the RNA extraction and RT-qPCR used (Supplementary Table 2).

### Detection Limit and Efficiency of the Procedure to Concentrate HEV in Drinking Water

**FIGURE 4:**
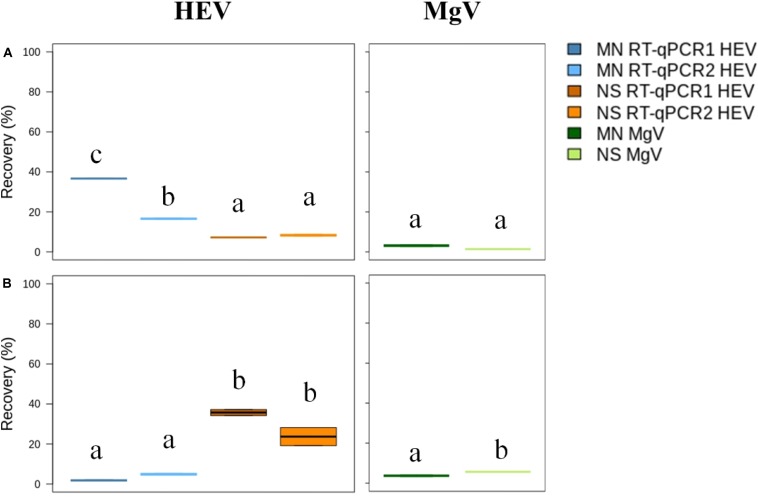
Median HEV recovery (%) in drinking water by Rexeed 25AX ultrafiltration followed by precipitation with polyethylene glycol **(A)** or centrifuge filtration with Amicon filters **(B)**. MN: NucleoSpin^®^RNA virus kit (Macherey-Nagel GmbH & Co.); NS: NucliSENS^®^ miniMag^®^ system (BioMérieux SA); RT-qPCR1: Schlosser et al., 2014; RT-qPCR2: ceeramTOOLS^®^ Hepatitis E Virus Detection KHEV kit (BioMérieux SA). Within each virus, different letters denote significant differences among methods (*P* < 0.05).

Prior to concentration, 20 L tap water samples were added with four different concentrations of HEV, and MgV, as a whole process control. Primary virus concentration was performed using DEUF with Rexeed-25A filters, resulting in an average eluate volume of 605 ± 38.22 mL. Then, the secondary concentration was evaluated comparing in parallel a PEG precipitation and a centrifuge filtration. DEUF ultrafiltration combined with PEG precipitation and MN kit resulted in HEV mean recovery of 16.6 to 36.6%, while recoveries ranged from 7.2 to 8.3% for NS kit (Figure 4A and Supplementary Table 3). The centrifuge filtration procedure using MN and NS resulted in mean HEV recovery values ranging from 1.8 to 4.9% and 23.7 to 35.7%, respectively (Figure 4B and Supplementary Table 4). A minimum recovery rate of 1% MgV was achieved from all procedures, validating the results. For all the tested procedures, the HEV LoD_95__%_ in drinking water was of 6.2 × 10^3^ IU/L (Supplementary Tables 3, 4).

### Performance of RT-qPCR Assays of HEV in Naturally Contaminated Wastewater Samples

**FIGURE 5:**
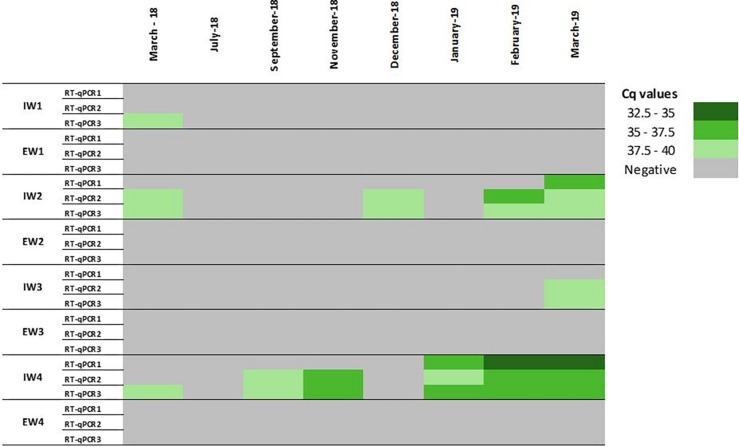
Occurrence of HEV in influent (IW) and effluent (EW) waters by comparing three RT-qPCR assays. RT-qPCR1: Schlosser et al. (2014); RT-qPCR2: ceeramTOOLS Hepatitis E Virus Detection KHEV kit (BioMérieux SA); RT-qPCR3: Girón-Callejas et al. (2015) (cf., Supplementary Table 5 for details of assays).

A lack of information on HEV viral loads before and after treatments applied in WWTPs has been identified (Fenaux et al., 2019). In the present study, a total of 64 samples were collected upstream (*n* = 32) and downstream (*n* = 32) of four WWTPs, and these samples were concentrated according to the Al procedure combined with the MN kit and analyzed by different RT-qPCRs assays. To improve the sensibility of the RT-qPCR assays, the initial 35 mL influent water sample volume was increased to 200 mL. Initially, two different RT-qPCRs were applied to assess HEV occurrence. Surprisingly, RT-qPCR1 showed a limited number of positives compared to RT-qPCR2 (Figure 5) despite a previous study reported similar performance of these assays in influent water samples (Randazzo et al., 2018). Suspecting that a different HEV genotype was circulating, a third RT-qPCR assay was included in the study. In particular, a method widely used in clinical and environmental virology firstly described by Jothikumar et al. (2006) and modified by Girón-Callejas et al. (2015) (RT-qPCR3) was applied to retest samples. All the samples had a minimum recovery rate of 1% MgV, validating the results. Overall, out of 32 influent water samples, 12 were positive for at least one of the three HEV RT-qPCR assays, and an overall HEV prevalence of 37.5% was found. Different numbers of positive samples and different prevalence rates were recorded during the comparison of the three RT-qPCR assays (Figure 5). In particular, prevalence rates of 12.5, 28.5, and 37.5% in influent waters were recorded for the RT-qPCR1, RT-qPCR2, and RT-qPCR3 assays, respectively. Although RT-qPCR1 fail to detect HEV in several samples, lower Cq values were observed in samples collected from January 2019 on (Figure 5). The observed differences may be due to HEV genotype variability. Unfortunately, conducted genotyping analyses did not solve the question because of the negative results, likely due to the low genome titers in the samples. Therefore, due to the high variability of the HEV genotypes (Smith et al., 2013, 2016), the RT-qPCR assays used for environmental analyses must be carefully checked to avoid false negative results.

### Surveillance of HEV in Influent and Effluent Water Samples From WWTPs and DWTPs

Data about the occurrence of HEV in Spanish wastewaters are scarce, therefore the number of influent and effluent samples were expanded and 62 influent and 52 effluent water samples were analyzed by RT-qPCR3 (Table 2). In the current study HEV is widely disseminated (30.65%) in Valencian influent waters even though the prevalence rates among WWTPs varies widely (Supplementary Figure 1). For example, WWTP4 had a prevalence rate of 73.33% (11/15) using the RT-qPCR3 assay. As a public health concern, this WWTP receives domestic sewage from several municipalities, accounting for 108,000 inhabitants, even though we cannot exclude streams from pig farms or other agricultural run-offs. In contrast, WWTP1 (14,000 inhabitants) and WWTP2 (188,000 inhabitants) showed only 12.5 and 13.33% prevalence, respectively. These data show no correlation between HEV prevalence and the number of inhabitants served by WWTPs (ρ_S_ = 0.26).

**TABLE 2:** Prevalence and HEV loads (IU/L) from four WWTPs (*n* = 114) and two drinking water treatment plants (*n* = 56) using RT-qPCR3.

**Type of water sample**	**Treatment plant**	**No of samples analyzed**	**No of positive samples**	**HEV prevalence (%)**	**Viral load(log IU/L) (range: min–max)**
Influent	WWTP1	16	2	12.50	3.11–3.57
	WWTP2	16	4	25.00	3.11–3.82
	WWTP3	15	2	13.33	3.11–3.79
	WWTP4	15	11	73.33	3.11–4.31
Effluent	WWTP1	13		0	ND
	WWTP2	13		0	ND
	WWTP3	13		0	ND
	WWTP4	13		0	ND
Influent	DWTP1	14		0	ND
	DWTP2	14		0	ND
Effluent	DWTP1	14		0	ND
	DWTP2	14		0	ND

*ND, not detected (below of limit of detection). WWTP, wastewater treatment plant; DWTP, drinking water treatment plant.*

Studies conducted in Barcelona (Spain) have shown similar prevalence (from 13.5 to 43.5% in influent waters, with absence or low detection of HEV in effluent waters (Clemente-Casares et al., 2003; Rodriguez-Manzano et al., 2010; Rusiñol et al., 2015).

The present study showed HEV contamination in influent waters ranging from approximately 1.3 × 10^3^–3.5 × 10^4^ IU/L using the RT-qPCR3 assay (Table 2), which is consistent with previously reported levels (Fenaux et al., 2019). HEV genomes were not detected in effluent waters (Table 2). These results are consistent with most of the studies published in Europe (Fenaux et al., 2019), even those done after a confirmed outbreak (Miura et al., 2016). This suggests that treatments applied at WWTPs (Supplementary Figure 1) are efficient in removing HEV despite the fact that a reduction of 1–2 log would result in concentrations below the LoD_95__%_. Thus, further improvements are needed to increase the sensitivity of the methods applied for virus concentration in effluent waters.

Additionally, a total of 56 samples were collected upstream (*n* = 28) and downstream (*n* = 28) of two DWTPs, and 20 L water samples were concentrated by DEUF using Rexeed-25A filters combined with PEG precipitation, the MN kit and analyzed by RT-qPCR3. None of the influent and effluent samples were positive for HEV despite all the samples had a minimum recovery rate of 1% MgV (Table 2).

## Conclusion

Hepatitis E virus is considered an emerging pathogen in industrialized countries, especially in Europe, and analytical procedures for estimating HEV concentrations in water samples are required. Among the different methodologies evaluated in this study, HEV concentration with aluminum hydroxide was able to detect HEV in influent and effluent water samples. However, the limited sensitivity of the method could be improved, for example by increasing the sample volume. The procedure for drinking water includes a DEUF step using a 30 kDa membrane to reduce the sample volume from 20 to 200 L to approximately 500 mL. Overall, the results showed that HEV is efficiently recovered from spiked drinking water samples processed using a PEG secondary concentration and the MN extraction kit.

This study also confirms that the selection of the RT-qPCR assays is critical since the overall performance of the methods varied considerably, most likely based on the circulating strains. In particular, this aspect remarkably affects genotyping results and thus epidemiology and traceability investigations.

Wastewater is an important environmental source for studying the epidemiology of viral pathogens transmitted via the fecal-oral route, and the current study demonstrated that HEV circulated in the Valencian region at around 30.6% during 2018–2019. No HEV was detected in effluent samples from WWTP and influent and effluent samples from DWTP. However, given that the infective dose in waterborne epidemics settings is not yet known and the low sensibility of the assay, unfortunately, no direct conclusion could be achieved on the risk assessment of environmental contamination.

## Data Availability Statement

All datasets generated for this study are included in the article/Supplementary Material.

## Author Contributions

EC-F, WR, and AP-C performed the assays, compiled the data, and wrote the draft of the manuscript. WR and GS conceived the original idea, interpreted the results, and drafted the manuscript. All authors contributed to the final manuscript.

## Conflict of Interest

The authors declare that the research was conducted in the absence of any commercial or financial relationships that could be construed as a potential conflict of interest.

## Footnotes

1 http://www.who.int/mediacentre/factsheets/fs280/en/

2 https://www.cdc.gov/hepatitis/hev/hevfaq.htm
