# Supplementary material for: HEV Occurrence in Waste and Drinking Water Treatment Plants
Source: Front Microbiol. 2020 Jan 14;10:2937. doi: 10.3389/fmicb.2019.02937 (PMC6971180; doi:10.3389/fmicb.2019.02937)

Supplementary material

**HEV occurrence in waste and drinking water treatment plants**

Enric Cuevas-Ferrando, Walter Randazzo, Alba Pérez-Cataluña, Gloria Sánchez

**Supplementary Table 1.** Limit of detection of HEV in influent wastewater samples.

| Extraction method | RT-qPCR | Levels of inoculated HEV  (IU/ 0.035L) | | | | LoD_95%_  (IU/L) | Mean mengovirus recovery  (min-max)  (%) |
| --- | --- | --- | --- | --- | --- | --- | --- |
|  |  | ≈1 x 10^5^ | ≈1 x 10^4^ | ≈1 x 10^3^ | ≈1 x 10^2^ |  |  |
| MN | RT-qPCR1 | 4/4^*^ | 4/4 | 4/4 | 0/4 | 3.57 x 10^4^ | 8.34A  (7.92 – 8.72) |
|  |  | 8.81^**^A  (3.57 – 12.27) |  |  |  |  |  |
|  | RT-qPCR2 | 4/4 | 3/4 | 1/4 | 0/4 | 5.43 x 10^5^ |  |
|  |  | 36.81A, B  (23.34 – 49.64) |  |  |  |  |  |
| NS | RT-qPCR1 | 4/4 | 0/4 | 0/4 | 0/4 | 4.29 x 10^6^ | 21.56B  (17.76 – 24.29) |
|  |  | 41.45B  (20.39– 81.22) |  |  |  |  |  |
|  | RT-qPCR2 | 4/4 | 4/4 | 3/4 | 0/4 | 7.57 x 10^4^ |  |
|  |  | 8.90A  (1.12 – 15.81) |  |  |  |  |  |

LoD_95_: limit of detection calculated according to Wilrich and Wilrich (2009); Within each column, different letters denote significant differences among methods (P < 0.05); ^*^HEV positive/total numbers of samples; ^**^Mean HEV recovery (min-max) (%).

**Supplementary Table 2.** Limit of detection of HEV in effluent water samples using the aluminum protocol.

| Extraction method | RT-qPCR | Levels of inoculated HEV  (IU/ 0.2 l) | | | | LoD_95%_  (IU/L) | Mean mengovirus recovery  (min-max)  (%) |
| --- | --- | --- | --- | --- | --- | --- | --- |
|  |  | ≈2 x 10^5^ | ≈2 x 10^4^ | ≈2 x 10^3^ | ≈2 x 10^2^ |  |  |
| MN | RT-qPCR1 | 4/4^*^ | 4/4 | 4/4 | 0/4 | 1.25 x 10^4^ | 41.17A  (30.08 – 54.50) |
|  |  | 30.01^**^A, B  (15.48 – 46.85) |  |  |  |  |  |
|  | RT-qPCR2 | 4/4 | 4/4 | 4/4 | 0/4 | 1.25 x 10^4^ |  |
|  |  | 8.33A  (2.55 – 18.53) |  |  |  |  |  |
| NS | RT-qPCR1 | 4/4 | 4/4 | 4/4 | 0/4 | 1.25 x 10^4^ | 23.90B  (21.52 – 28.32) |
|  |  | 7.72A  (4.84 –11.85) |  |  |  |  |  |
|  | RT-qPCR2 | 4/4 | 4/4 | 4/4 | 0/4 | 1.25 x 10^4^ |  |
|  |  | 41.90B  (34.19 – 54.85) |  |  |  |  |  |

LoD_95_: limit of detection calculated according to Wilrich and Wilrich (2009); Within each column, different letters denote significant differences among methods (P < 0.05); ^*^HEV positive/total numbers of samples; ^**^Mean HEV recovery (min-max) (%).

**Supplementary Table 3.** Limit of detection of HEV in tap water by Rexeed 25AX ultrafiltration followed by precipitation with polyethylene glycol.

| Extraction method | RT-qPCR | Levels of inoculated HEV  (IU/ 20 l) | | | | LoD_95%_  (IU/L) | Mean mengovirus recovery  (min-max)  (%) |
| --- | --- | --- | --- | --- | --- | --- | --- |
|  |  | ≈1 x 10^7^ | ≈1 x 10^6^ | ≈1 x 10^5^ | ≈1 x 10^4^ |  |  |
| MN | RT-qPCR1 | 4/4^*^ | 4/4 | 4/4 | 0/4 | 6.2 x 10^3^ | 3.14A  (2.65 – 3.63) |
|  |  | 36.65^**^C  (36.39-36.90) |  |  |  |  |  |
|  | RT-qPCR2 | 4/4 | 4/4 | 4/4 | 0/4 | 6.2 x 10^3^ |  |
|  |  | 16.59B  (16.24-16.94) |  |  |  |  |  |
| NS | RT-qPCR1 | 4/4 | 4/4 | 4/4 | 0/4 | 6.2 x 10^3^ | 1.39A  (1.34 – 1.45) |
|  |  | 7.24A  (7.18-7.29) |  |  |  |  |  |
|  |  | 4/4 | 4/4 | 4/4 | 0/4 | 6.2 x 10^3^ |  |
|  | RT-qPCR2 | 8.33A  (7.86-8.80) |  |  |  |  |  |

LoD_95_: limit of detection calculated according to Wilrich and Wilrich (2009); Within each column, different letters denote significant differences among methods (P < 0.05); ^*^HEV positive/total numbers of samples; ^**^Mean HEV recovery (min-max) (%).

**Supplementary Table 4.** Limit of detection of HEV in tap water by Rexeed 25AX ultrafiltration follow by centrifuge filtration with Amicon filters.

| Extraction method | RT-qPCR | Levels of inoculated HEV  (IU/ 20 l) | | | | LoD_95%_  (IU/L) | Mean mengovirus recovery  (min-max)  (%) |
| --- | --- | --- | --- | --- | --- | --- | --- |
|  |  | ≈1 x 10^7^ | ≈1 x 10^6^ | ≈1 x 10^5^ | ≈1 x 10^4^ |  |  |
| MN | RT-qPCR1 | 4/4^*^ | 4/4 | 4/4 | 0/4 | 6.2 x 10^3^ | 3.72A  (3.27 – 4.17) |
|  |  | 1.82^**^A  (1.45-2.19) |  |  |  |  |  |
|  | RT-qPCR2 | 4/4 | 4/4 | 4/4 | 0/4 | 6.2 x 10^3^ |  |
|  |  | 4.90A  (4.38-5.41) |  |  |  |  |  |
| NS | RT-qPCR1 | 4/4 | 4/4 | 4/4 | 0/4 | 6.2 x 10^3^ | 5.72B  (5.64 – 5.79) |
|  |  | 35.70B  (34.19-37.21) |  |  |  |  |  |
|  | RT-qPCR2 | 4/4 | 4/4 | 4/4 | 0/4 | 6.2 x 10^3^ |  |
|  |  | 23.69B  (19.15-28.23) |  |  |  |  |  |

LoD_95_: limit of detection calculated according to Wilrich and Wilrich (2009); Within each column, different letters denote significant differences among methods (P < 0.05)^*^HEV positive/total numbers of samples; ^**^Mean HEV recovery (min-max) (%).

**Supplementary Table 5.** List of primers and probes used in this study for HEV analysis.

| **Assay** | **Amplification region** | **Primers and probe** | **Sequence 5'-3'** | **RT-qPCR conditions** | **Location*** | **Reference** |
| --- | --- | --- | --- | --- | --- | --- |
|  |  |  |  |  |  |  |
|  |  |  |  |  |  |  |
| RT-qPCR1 | ORF3 | HEV.Fa | GTGCCGGCGGTGGTTTC | RT 50 °C for 30’ | 5296–5377 (81 nt) | Schlosser et al. (2014) with modified probe |
|  |  | HEV.Fb | GTGCCGGCGGTGGTTTCTG | 95 °C for 15’ |  |  |
|  |  | HEV.R | GCGAAGGGGTTGGTTGGATG | PCR (45x) |  |  |
|  |  | HEV.P | FAM-TGACMGGGT/ZEN/TGATTCTCAGCC/3IABkFQ | 95 °C for 10'' |  |  |
|  |  |  |  | 55 °C for 20'' |  |  |
|  |  |  |  | 72 °C for 15'' |  |  |
|  |  |  |  |  |  |  |
| RT-qPCR2 | ORF3 | N/A | N/A | RT 45 °C for 10’ | N/A | Ceeram (hepatitis@ceeramTools) |
|  |  |  |  | 95 °C for 10’ |  |  |
|  |  |  |  | PCR (40x) |  |  |
|  |  |  |  | 95 °C for 15'' |  |  |
|  |  |  |  | 60 °C for 45'' |  |  |
|  |  |  |  |  |  |  |
| RT-qPCR3 | ORF3 | JVHEVF | GGTGGTTTCTGGGGTGAC | RT 50 °C for 30’ | 5304–5373 (69 nt) |  |
|  |  | JVHEVRmod | AGGGGTTGGTTGGRTGRA | 95 °C for 2’ |  | Jothikumar et al. (2006) modified; |
|  |  | JVHEVPmod | TGATTCTCAGCCCTTCGC | PCR (45x) |  | Girón-Callejas, Clark, Irving, & McClure (2015) |
|  |  |  |  | 95 °C for 15'' |  |  |
|  |  |  |  | 60 °C for 40'' |  |  |

**^⁎^** Location in reference to WHO International Standard for HEV RNA, HRC-HE104 strain, accession no. AB630970 (Baylis et al., 2013).

**Supplementary Figure 1.** Reclamation processes applied in the four wastewater treatment plants selected in this study.


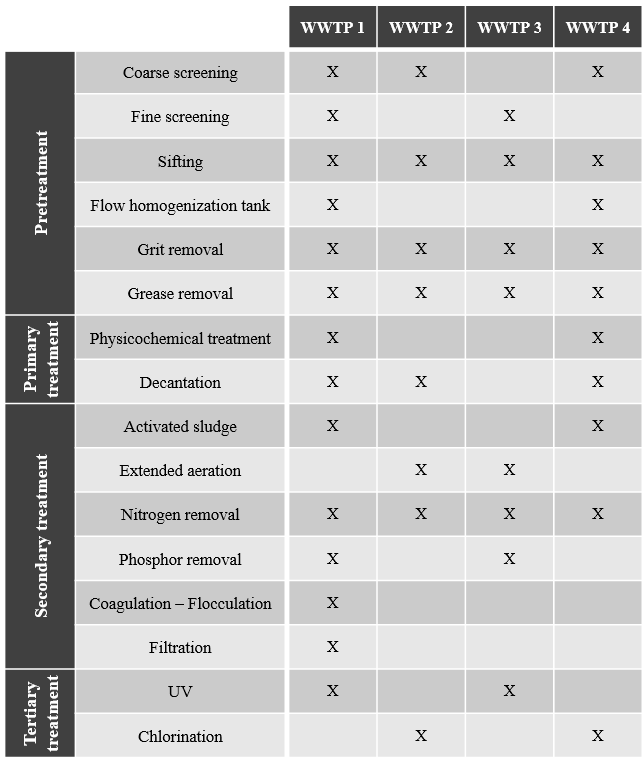

Supplement: Supplementary file 1 [file Data_Sheet_1.docx]
